# Laparotomy in Ectopic Pregnancy

**Published:** 1890-11

**Authors:** Wm. H. Wathen

**Affiliations:** Louisville, Ky.


### Southern Medical Record.

A MONHLY JOURNAL OF MEDICINE AND SURGERY.

VOL. XX. ATLANTA, GA., NOVEMBER, 1890. NO. 11.

### Original Articles.

### LAPAROTOMY IN ECTOPIC PREGNANCY.

BY WM. H. WATHEN, M. D., LOUISVILLE, KY.

An Abstract of a paper read by Dr. Wathen, of Louisville, before the Tri-State Medical Society of Tennessee, Alabama and Georgia, Oct. 15, 1890.

### LAPAROTOMY VS. ELECTRICITY IN ECTOPIC PREGNANCY.

Dr. Wathen said: Electricity, the only feticidal means now recognized as orthodox by physicians who practice destroying the life of the fetus in ectopic pregnancy, is no longer used for this purpose where the pregnancy has continued beyond three and a half or four months, and is seldom used after the third month. At this time the fetus cannot be killed except by electro-puncture, and the complications and the deaths consequent to this practice have been so numerous that the most radical advocates of electricity are afraid to introduce the electrodes into the gestation sac. The use of electricity in extra-uterine pregnancy is practically confined to the United States, and while it is advocated by men of recognized ability and learning in obstetrics and gynecology, I am constrained to believe that very soon it will have no support.

The immediate and subsequent results of electricity as a feticide are put in the most favorable attitude in a paper by Dr. Brothers, in the February, 1890, issue of the *American Journal of Obstetrics and Diseases of Women and Children.*

Every fact supposed to favor its use is made to sound its praise extravagantly, but the many unfortunate results that speak volumes against its use are quietly passed by, or an effort made to brush them aside. Still, the conclusions of the author furnish the strongest proof in favor of laparotomy.

These statistics, compared with the statistics of laparotomy, show conclusively that the use of electricity in extra-uterine pregnancy is more dangerous, granting that there was an error in diagnosis.

But just here we have another argument in favor of laparotomy, for the difficulty, and sometimes the impossibility, of diagnosticating extra-uterine pregnancy in the early months, is so manifest to experienced physicians, that it would be ridiculous to claim that in all these cases pregnancy existed, while in the cases where laparotomy is done a diagnosis may positively be made by seeing the embryo or the chorionic or placental villi. If the embryo or fetus in an extra-uterine pregnancy is killed by electricity, a more or less diseased condition of the pelvic structures is left that endangers the health or the life of the woman—the dangers usually being increased as pregnancy advances. But if a laparotomy is done, there is no obstructed tube, or other pathological condition left, and if the woman recovers from the immediate effects of the operation she is entirely cured. Her life is no longer in jeopardy because of the danger of pelvic abscess, sepsis, or exhaustion following an effort to discharge the suppurating contents of the gestation sac through fistulous tracts into the rectum, vagina, bladder, or through the abdominal walls. If we could eliminate the cases where there was an error in diagnosis, we would find that the mortality from the use of electricity and the after results are far in excess of what follows laparotomy in the practice of experienced operators.

In an examination of Brothers' report of the subsequent behavior of twenty-five cases observed at periods varying from one to eight years after the employment of electricity, we must be impressed with the fact that at least fifty per cent. of all cases carefully observed had thickening or distinct tumor, that may at any time require laparotomy to save the woman's life, or to cure her of confirmed invalidism.

In cases where laparotomy has been done, the mortality has not exceeded five per cent., and nearly all the women that recovered from the immediate effects of the operation were permanently cured. Most of these operations were done after rupture of the sac, where the conditions are not so favorable in laparotomy work.

Laparotomy, in all cases where the advocates of electricity would defend its use, is so simple and so free of danger that I believe an experienced operator could save at least ninety-five per cent. of his patients, probably ninety-nine per cent.

And before the end of the third month there is no condition requiring laparotomy where the operation is more easily done or the immediate or subsequent dangers fewer. In fact, its simplicity, compared with the operation for many pathological conditions in the pelvis, is so decided that in the practice of a successful laparotomist, who observes the rules of clean surgery, and adopts the most approved technique, the patients should recover. Tait's operations have been for ruptured tubal pregnancy, and he reports but two deaths in more than fifty patients. The first woman he operated on died because he did not then know the correct technique in such cases, and his second death was where the woman was in fatal shock before the operation.

One of the reasons given in favor of electricity is, that these women cannot always be operated on by an experienced laparotomist. Nor can electricity be always used by men who are familiar with its successful use in such cases. If it is necessary to refer the woman to some specialist in laparotomy, it will as often be necessary to refer her to some specialist in electricity, who has all the appliances necessary for good results in such work.

The services of an experienced abdominal surgeon may be obtained as easily as the services of a man experienced in the successful use of electricity. The operation is so similar to the operation for the removal of enlarged and slightly adherent ovaries, that a description of it would be unnecessary. If the sac has become adherent to the pelvic structure, it should be gently but quickly separated and ligated at its base with a double ligature, being careful to include both the distal and proximal ends of the vessels.

In conclusion, I wish to show you two specimens which will illustrate some of the phases of extra-uterine pregnancy. The first is a placenta with the fetus attached, showing the ovaries and tubes successfully removed by me some months ago. This is a tubal pregnancy that ruptured into the folds of the broad ligament. The second is a uterus with the adnxea showing a ruptured tubal pregnancy into the abdomen at about the sixth week. This was removed post mortem by Dr. Kelch. The rupture occurred thirty-six hours before death. Dr. Keleh made a correct diagnosis soon after the rupture, and urged the patient and her family to allow a laparotomy. This was refused until an hour before her death, and when I entered her room she was dying. Her life could have been saved had she consented to an early operation.

